# Vertebral Osteomyelitis Caused by *Mycobacterium arupense* Mimicking Tuberculous Spondylitis: First Reported Case and Literature Review

**DOI:** 10.1093/ofid/ofad019

**Published:** 2023-01-17

**Authors:** Ayu Kasamatsu, Kazuaki Fukushima, Yuriko Igarashi, Satoshi Mitarai, Yuka Nagata, Masao Horiuchi, Noritaka Sekiya

**Affiliations:** Department of Infectious Diseases, Tokyo Metropolitan Cancer and Infectious Diseases Center Komagome Hospital, Tokyo, Japan; Department of Infection Prevention and Control, Tokyo Metropolitan Cancer and Infectious Diseases Center Komagome Hospital, Tokyo, Japan; Department of Infectious Diseases, Tokyo Metropolitan Cancer and Infectious Diseases Center Komagome Hospital, Tokyo, Japan; Department of Mycobacterium Reference and Research, Research Institute of Tuberculosis, Japan Anti-Tuberculosis Association, Tokyo, Japan; Department of Mycobacterium Reference and Research, Research Institute of Tuberculosis, Japan Anti-Tuberculosis Association, Tokyo, Japan; Department of Infection Prevention and Control, Tokyo Metropolitan Cancer and Infectious Diseases Center Komagome Hospital, Tokyo, Japan; Department of Infection Prevention and Control, Tokyo Metropolitan Cancer and Infectious Diseases Center Komagome Hospital, Tokyo, Japan; Department of Infection Prevention and Control, Tokyo Metropolitan Cancer and Infectious Diseases Center Komagome Hospital, Tokyo, Japan; Department of Clinical Laboratory, Tokyo Metropolitan Cancer and Infectious Diseases Center Komagome Hospital, Tokyo, Japan

**Keywords:** *Mycobacterium arupense*, mycobacterium infections, nontuberculous mycobacteria, osteomyelitis, spondylitis

## Abstract

*Mycobacterium arupense* is a slow-growing, nontuberculous mycobacterium widely found in the environment and is known to cause tenosynovitis and osteomyelitis, mainly in the hands and wrists. We present the first case of vertebral osteomyelitis caused by *M arupense* in a 78-year-old man with renal cell carcinoma. The patient had a history of tuberculous pleuritis in childhood. Although the nucleic acid amplification test of the vertebral tissue for *Mycobacterium tuberculosis* was negative, we initiated tuberculosis treatment based on the history and pathological findings of auramine-rhodamine-positive organisms and epithelioid cell granulomas. Subsequently, the isolated mycobacterium was identified as *M arupense* by genome sequencing. Accordingly, the treatment regimen was changed to a combination of clarithromycin, ethambutol, and rifabutin. Owing to a subsequent adverse event, rifabutin was switched to faropenem, and the patient was treated for a total of 1 year. In previous literature, we found 15 reported cases of bone and soft tissue infections caused by *M arupense*, but none of them had vertebral lesions. Physicians should be aware that *M arupense* can cause vertebral osteomyelitis mimicking tuberculous spondylitis. In addition, molecular testing of isolated mycobacteria is essential for diagnosis, even if tuberculous spondylitis is suspected.

*Mycobacterium arupense* is a slow-growing nontuberculous mycobacterium (NTM) that was first isolated from clinical specimens in 2006 [1]. Since then, only a few cases of bone and soft tissue infections caused by *M arupense* have been reported. Here, we report the first case of vertebral osteomyelitis caused by *M arupense*, along with a literature review of bone and soft tissue infections caused by this pathogen.

## CASE REPORT

A 78-year-old Japanese man presented with lower back pain of 8 months’ duration and bilateral leg pain of 1 month’s duration. He had developed tuberculous pleuritis at 11 years of age. The patient had no history of trauma. His mother had a history of tuberculosis when he was 7 years old. He had no occupational or avocational exposure to wet soil or water. He had never traveled abroad.

**Figure 1.**
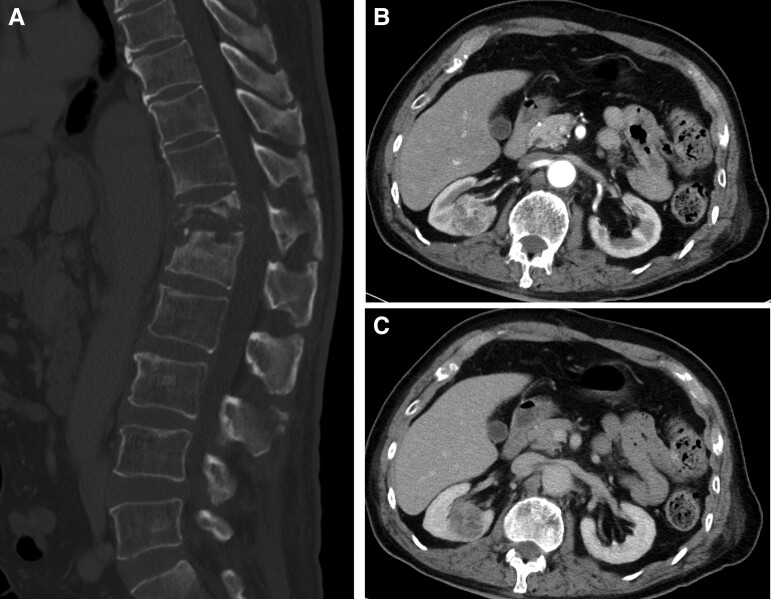
Contrast-enhanced computed tomographic scan showed a pathological fracture and osteolytic changes in the vertebra at the T12–L1 levels. A mass lesion with early enhancement was observed in the right kidney. *A*, Sagittal. *B*, Axial, early arterial phase. *C*, Axial, late phase.

**Figure 2.**
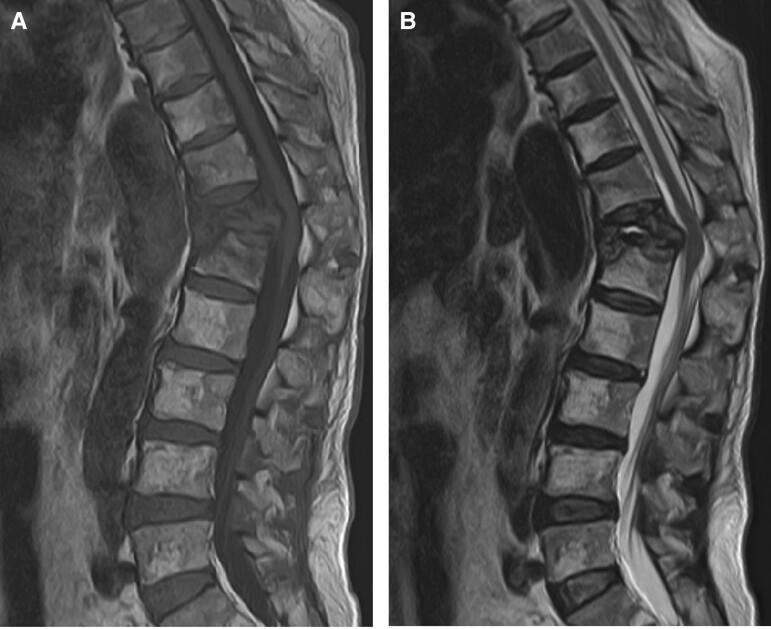
Magnetic resonance imaging showed the T12–L1 vertebral fractures and their advance into the spinal canal. *A*, Sagittal, T1-weighted. *B*, Sagittal, T2-weighted.

At the presentation, the patient's vital signs were normal. Physical examination revealed an increased patellar tendon reflex on the left side and bilaterally decreased Achilles tendon reflexes. He was capable of bending both knees and moving both ankles as intended. Blood tests showed normal white blood cell counts and elevated C-reactive protein level (6.6 mg/dL) and erythrocyte sedimentation rate (55 mm/hour). Mild renal dysfunction was observed, with a creatinine of 1.35 mg/dL. The soluble interleukin-2 receptor level was elevated to 764 U/mL. T-SPOT.TB (Oxford Immunotec) and anti–human immunodeficiency virus antigens/antibodies tested negative. Computed tomography revealed a fracture with osteolytic changes in Th12–L1 vertebrae and a mass in the right kidney (Figure 1). Magnetic resonance imaging revealed Th12–L1 vertebral fractures and protrusion of the vertebrae into the spinal canal (Figure 2).

**Figure 3.**
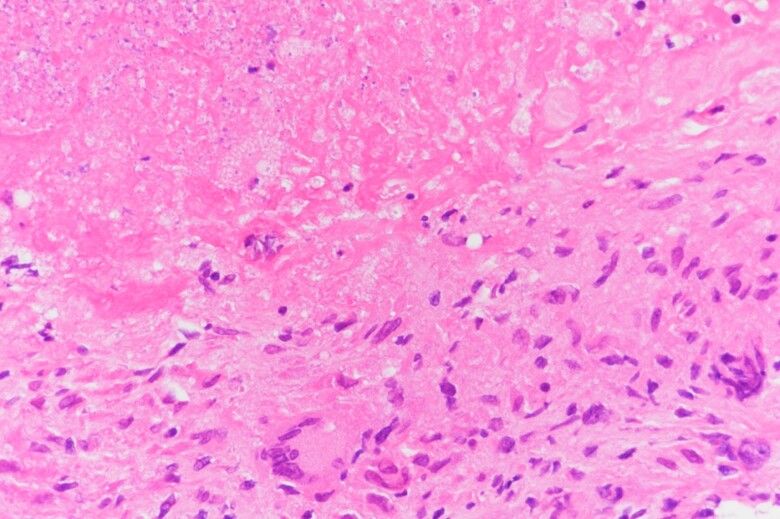
Pathological findings of the vertebral tissue. Caseous necrosis surrounded by epithelial granulomas and multinucleated Langhans giant cells were observed.

**Figure 4.**
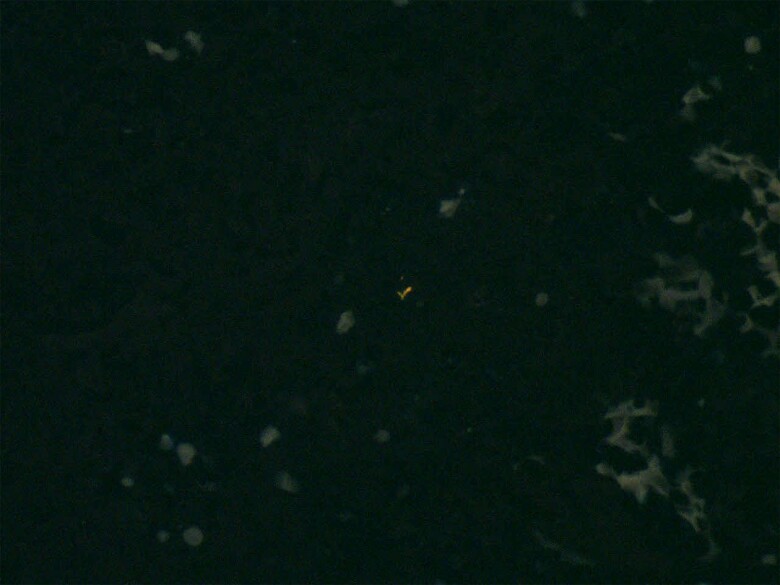
Pathological findings of the vertebral tissue. Auramine-rhodamine staining revealed acid-fast bacilli.

Three days after presentation, he underwent posterior vertebral decompression and percutaneous thoracolumbar root screw fixation for neurological deficits and vertebral biopsy for a pathological vertebral fracture. No remarkable abscess or necrotic tissue was observed intraoperatively. Vertebral tissue samples were negative for general bacterial culture, concentrated fluorochrome smear microscopy, and nucleic acid amplification testing for *Mycobacterium tuberculosis*. Pathological examination of the vertebral tissue showed caseous necrosis surrounded by epithelial granulomas and a few multinucleated Langhans giant cells (Figure 3). A few acid-fast bacilli were found on auramine-rhodamine and acid-fast stains; no neoplastic lesions were detected (Figure 4). Based on these findings, the patient was presumed to have tuberculous spondylitis, and oral rifampicin, isoniazid, ethambutol (EMB), and pyrazinamide administration was commenced on the eighth postoperative day.

Bacteriological investigation revealed the growth from vertebral tissue cultures on 2% Ogawa medium (Kyokuto Pharmaceuticals) in the second week at 30°C and in the third week at 37°C, respectively. Cultures on a liquid medium (Mycobacteria Growth Indicator Tube, Becton Dickinson) at 37°C were negative for 12 weeks. Bacteria and mycobacteria were not isolated from sputum or urine cultures. *Mycobacterium tuberculosis* group antigen or mycobacterial protein fraction from bacille Calmette–Guérin of Rm 0.64 in electrophoresis tested negative. Matrix-assisted laser desorption/ionization time-of-flight mass spectrometry indicated an NTM suggestive of *M arupense* infection. Based on these results, pyrazinamide was switched to clarithromycin (CLR) on postoperative day 27. Subsequent identification tests revealed 100% homology with *M arupense* on 16S ribosomal RNA (rRNA), 99.75% on *hsp*65, and 98.91% on *rpoB* gene sequencing. The relationship between 16S rRNA gene sequence of this isolate and that of other species of *Mycobacterium terrae* complex (MTC) is shown in the Supplementary Figure. Drug susceptibility testing showed that the isolate was susceptible to rifabutin (RFB), EMB, and CLR based on the interpretation for *Mycobacterium kansasii* of the Clinical and Laboratory Standards Institute document M62 (Table 1) [2]. The regimen was then adjusted to 3-drug combination therapy of RFB, CLR, and EMB on postoperative day 126. Subsequently, neutropenia and thrombocytopenia, likely due to the interaction between RFB and CLR, were observed, and RFB was switched to faropenem (FRPM) on a postoperative day 160. Antimicrobial therapy was completed 1 year after the initiation of CLR treatment. His symptoms gradually improved, and his condition has been stable for 2 years after treatment. A partial nephrectomy was performed for renal cancer.

**Table 1.** Results of Antimicrobial Susceptibility Testing for the Isolate of *Mycobacterium arupense*

Antimicrobial Drug	MIC, μg/mL
Amikacin	>128
Azithromycin	32
Ciprofloxacin	>64
Clarithromycin	1
Clofazimine	2
Doxycycline	>16
Ethambutol	0.5
Faropenem	≤2
Imipenem	>64
Isoniazid	>64
Kanamycin	>128
Levofloxacin	>32
Linezolid	>32
Meropenem	64
Moxifloxacin	>8
Rifabutin	0.25
Rifampicin	16
Sitafloxacin	>8
Streptomycin	>128
Sulfamethoxazole-trimethoprim	>152/8
Tobramycin	>16

Abbreviation: MIC, microdilution interpretive criteria.

## DISCUSSION AND LITERATURE REVIEW

To the best of our knowledge, this is the first reported case of vertebral osteomyelitis caused by *M arupense* infection. The organism was first isolated from clinical specimens by Cloud et al in 2006 [1] and was classified as MTC, which are slow-growing NTM. Recent developments in genetic analysis have revealed that *M arupense*, like other bacteria of the MTC, is widely distributed in the environment, including in soil and surface water [3, 4]. MTC causes bone and soft tissue infections through direct exposure to environmental organisms [5]. Nevertheless, *M arupense* is a rare pathogen causing NTM infection in humans. We reviewed the English and Japanese literature on clinical cases of bone and soft tissue infections caused by this organism using PubMed, Google Scholar, and Ichushi-Web (a bibliographic database of articles published in Japanese-language medical journals). The search term was “*Mycobacterium arupense*.” Fifteen cases were identified, 13 of which were tenosynovitis or osteomyelitis of the fingers, hands, or wrists (Table 2) [6–20]. In addition to other clinical manifestations, pneumonitis and bacteremia have been reported [21, 22]. No previous cases of *M arupense* infection presenting with vertebral osteomyelitis have been reported. Additionally, this organism has not been isolated or identified from the vertebrae in retrospective molecular studies of clinical specimens [10, 23–25].

**Table 2.** Summary of Published Cases of Bone and Soft Tissue Infection Caused by *Mycobacterium arupense*

Reference	Publication Year	Age, Sex	Characteristics	Positive Specimen, Diagnosis	Treatment	Duration	Outcome
Tsai et al [6]	2008	54, F	Diabetes mellitus, history of traffic accident	Skin, hand tenosynovitis	Synovectomy, debridement, medication (CLR, EMB, MXF, RFB)	6 mo	Improved
Senda et al [7]	2011	68, M	Steroid injections	Synovium, hand tenosynovitis	Synovectomy, medication (EMB, RIF)	14 mo	Improved
Legout et al [8]	2012	35, M	History of hand injury and exposure to mud, steroid injection	Synovial fluid, wrist osteomyelitis	Synovectomy, medication (AMK, CLR, CIP, EMB, followed by CLR, CIP)	12 mo	Improved
Lee et al [11]	2014	56, F	History of puncture injury to the finger, steroid use, steroid injections	Resected tissue, hand tenosynovitis	Drainage, medication (CLR, EMB, RIF)	NA	Improved
Beam et al [10]	2014	58, M	History of blunt trauma to the finger, farmer	Tenosynovium, hand and wrist tenosynovitis	Synovectomy, medication (CLR, EMB, MXF, RIF, followed by CLR, EMB, RFB)	12 mo	Improved
Seidl & Lindeque [12]	2014	69, F	Traumatic joint arthrotomy, recurrent knee infection	Surgical culture, knee osteoarticular joint infection	Debridement, synovectomy, bone resection, medication (AZM, EMB, RIF)	4 mo	Improved
Ogawa et al [19]	2014	76, M	Cook	Tissue sample, forearm tenosynovitis	Debridement, synovectomy, medication (CLR, RIF, STR)	9 mo	Improved
Lopez et al [9]	2016	62, M	NK cell deficiency, hyper–IL-6 syndrome, recurrent polychondritis, Sweet syndrome; IVIG, steroid/canakinumab use	Skin, hand tenosynovitis	Synovectomy, medication (CLR, EMB, RFB)	12 mo	Improved
Yamamoto et al [20]	2020	80, F	Myelodysplastic syndrome, diabetes mellitus, polymyalgia rheumatica, steroid use	Synovium/synovial fluid, wrist tenosynovitis	Debridement, synovectomy, medication (LVX, RIF)	6 mo	Improved after temporary exacerbation
Navid et al [14]	2020	51, F	Diabetes mellitus, foot ulcer, farmer, HTLV-1 carrier	Ulcerative lesion, wound infection of the foot	Drainage, medication (CLR, EMB, RFB)	2 wk	Improved
Jaime-Villalonga et al [13]	2020	50, M	Posttraumatic cement spacer placement in the finger	Tissue sample, finger tenosynovitis and osteomyelitis	Drainage, hardware removal, medication (EMB, RFB, followed by CLR, EMB)	12 mo	Improved
Yokozeki et al [15]	2020	64, M	Car painter and bodyworker, history of finger injuries due to fishing	Aspiration sample, hand and wrist tenosynovitis	Debridement, synovectomy, medication (CLR, EMB, RIF)	2 y	Improved
Uehara et al [18]	2021	70, M	Rheumatoid arthritis, steroid/methotrexate use	Tenosynovium, hand and wrist tenosynovitis	Synovectomy, medication (CLR, EMB, RIF)	NA	Improved
Patel et al [16]	2021	50, M	Surgeon, history of splinter injury following gardening	Tenosynovial fluid/debris, hand tenosynovitis	Drainage, tenosynovectomy, medication (CLR, EMB, RFB ± AMK)	12 mo	Improved
Turner et al [17]	2021	60, M	Rheumatoid arthritis, infliximab/methotrexate use, history of finger injury	Synovium, hand tenosynovitis	Debridement, medication (AZM, EMB, RIF)	12 mo	Improved

Abbreviations: AMK, amikacin; AZM, azithromycin; CLR, clarithromycin; CIP, ciprofloxacin; EMB, ethambutol; F, female; HTLV-1, human T-cell lymphotrophic virus type 1; IL-6, interleukin 6; IVIG, intravenous immunoglobulin; LVX, levofloxacin; M, male; MXF, moxifloxacin; NA, not applicable; NK, natural killer; RFB, rifabutin; RIF, rifampicin; STR, streptomycin.

The patient was initially suspected of having tuberculous spondylitis based on the pathological findings of granuloma with caseous necrosis, auramine-rhodamine stain–positive organisms, and a family and personal history of tuberculosis. Generally, NTM is unlikely to be at the top of the differential list because of its rarity among the causative organisms of vertebral osteomyelitis [26]. Furthermore, the acid-fast bacilli smear test and culture of biopsy specimens cannot differentiate NTM from *M tuberculosis*. This makes molecular identification methods crucial when mycobacteria are detected in vertebral specimens, even when *M tuberculosis* is suspected. However, careful interpretation is needed to determine if the *M arupense* detected is the etiological cause, given the clinical presentation and the possibility of contamination. The pathogenicity of *M arupense* detected in respiratory specimens is unknown [27], especially in patients with cancer. A retrospective observational study reported comparable outcomes with and without antimicrobial therapy in patients with cancer in whom *M arupense* was isolated from respiratory specimens, indicating colonization [25]. We diagnosed this case as vertebral osteomyelitis caused by *M arupense* because it was detected in a vertebral lesion, and there was no strong evidence of a differential diagnosis, such as metastatic malignancy or tuberculosis.

Bone and soft tissue infections with *M arupense*, as with other NTM, predominately occur in patients with immunocompromised conditions (eg, immunosuppressive drug use and diabetes mellitus), history of injury, and activities that increase the risk of hand injury and environmental exposure (eg, farming and fishing) (Table 2). In contrast, NTM vertebral osteomyelitis is rarely caused by direct inoculation from an injury. This phenomenon is more commonly observed in immunocompromised individuals [28]. Our patient had no history of injury; however, he was found to have renal cancer, which may have been a predisposing factor. Little is known about the pathophysiology of NTM vertebral osteomyelitis in patients without a history of trauma. However, it has been hypothesized that NTM that colonize the respiratory or gastrointestinal tract mucosa are taken up by endocytosis when they come into contact with macrophages, which then mobilize to the site of bone formation and release the NTM, leading to localized osteomyelitis [26].

The optimal antimicrobial regimen and treatment duration for *M arupense* has not yet been established. There are no randomized controlled trials or comparative studies evaluating the management of osteomyelitis due to NTM; therefore, management relies on case reports, reviews of cases, drug susceptibility testing results, and official American Thoracic Society/Infectious Diseases Society of America statements [29]. However, combination antimicrobial therapy, often combined with surgical debridement and drainage of abscess, is commonly performed for localized vertebral osteomyelitis since it can serve as a storage site for NTM [26, 30]. In our patient, these surgical procedures were not performed because no abscess or necrotic tissue was observed during the decompression. We initiated a standard 4-drug combination therapy for tuberculosis because we initially considered a diagnosis of tuberculous spondylitis based on the histological findings and family history. Previous studies on the in vitro susceptibility of *M arupense* to antimicrobial agents have shown that it is often susceptible to amikacin, CLR, EMB, RFB, and sulfamethoxazole-trimethoprim but resistant to ciprofloxacin, levofloxacin, and rifampicin [5, 10, 23]. Thus, standard 4-drug therapy targeting *M tuberculosis* may be inappropriate. In addition, even if an isolate is susceptible to a particular drug in vitro, careful follow-up is crucial to ensure a clinical response. We treated our patient with CLR, EMB, and RFB after confirming that the strain was sensitive to these 3 drugs, which is consistent with many previous reports. Subsequently, we switched from RFB to FRPM due to the appearance of side effects; no previous cases of bone and soft tissue infections due to this pathogen were treated with FRPM (Table 2). Furthermore, although the effectiveness of FRPM against *M tuberculosis* and rapidly growing mycobacteria has been suggested in case reports [31–33], its effectiveness against slow-growing NTM has rarely been described. Given the susceptibility results and the successful clinical course of this case after treatment, FRPM may be considered an alternative drug candidate. Vertebral osteomyelitis due to NTM requires antimicrobial therapy for at least 4–6 months after therapeutic response. The duration may be considered a year or more in immunocompromised individuals or in those with inadequate debridement [26]. Previous cases of bone and soft tissue infections caused by *M arupense* were treated for approximately 1 year regardless of the immune status, and improvements were observed (Table 2). In accordance with these findings, we treated the patient for 1 year.

Bone and soft tissue infections caused by *M arupense* have a favorable prognosis in most cases (Table 2). Our patient also improved and had no recurrence.

## CONCLUSIONS

Herein, we presented the first case of vertebral osteomyelitis caused by *M arupense* in a patient with renal cancer. He improved after 1 year of antimicrobial therapy with no relapse. *M arupense* can cause vertebral osteomyelitis mimicking tuberculous spondylitis; therefore, greater emphasis should be placed on pathogen recovery and definitive molecular testing for the mycobacterium in the affected tissue.

## Supplementary Material

ofad019_Supplementary_Data

## Supplementary Data

Supplementary materials are available at *Open Forum Infectious Diseases* online. Consisting of data provided by the authors to benefit the reader, the posted materials are not copyedited and are the sole responsibility of the authors, so questions or comments should be addressed to the corresponding author.

## Notes

***Author contributions.*** Conception of the work, data collection, and literature review: A. K., K. F. Drafting the first manuscript: A. K. Critical revision of the manuscript: K. F., S. M., Y. I., Y. N., M. H., N. S. All authors approved the submitted manuscript version and have agreed to be personally accountable for any questions related to the accuracy or integrity of any part of the work.

***Acknowledgments.*** We thank Dr Masanori Fujiwara and all of the staff at the Tokyo Metropolitan Cancer and Infectious Diseases Center, Komagome Hospital, for their excellent patient care. We are also grateful to Dr Tsunekazu Hishima for providing pathological findings.

***Patient consent.*** The patient's written consent was obtained before publication of this report. All patient-specific information has been anonymized as much as possible. No human subject experiments were conducted related to this case report. Therefore, ethics committee approval was not sought.

***Financial support.*** There was no financial support received for this report.
