# Supplementary material for: Vertebral Osteomyelitis Caused by Mycobacterium arupense Mimicking Tuberculous Spondylitis: First Reported Case and Literature Review
Source: Open Forum Infect Dis. 2023 Jan 17;10(1):ofad019. doi: 10.1093/ofid/ofad019 (PMC9887264; doi:10.1093/ofid/ofad019)
Supplement: ofad019_Supplementary_Data [file ofad019_supplementary_data.docx]

**Supplementary Figure.** A phylogenetic tree generated by ClustalW based on 16S rRNA sequence analysis of our isolate (19048) and other isolates of the *Mycobacterium terrae* complex (AR30097, NTC13432, JCM12143, CST7274, MO-233) using the neighbor-joining method. The number under the line shows the absolute score of the differences in the sequence.

**
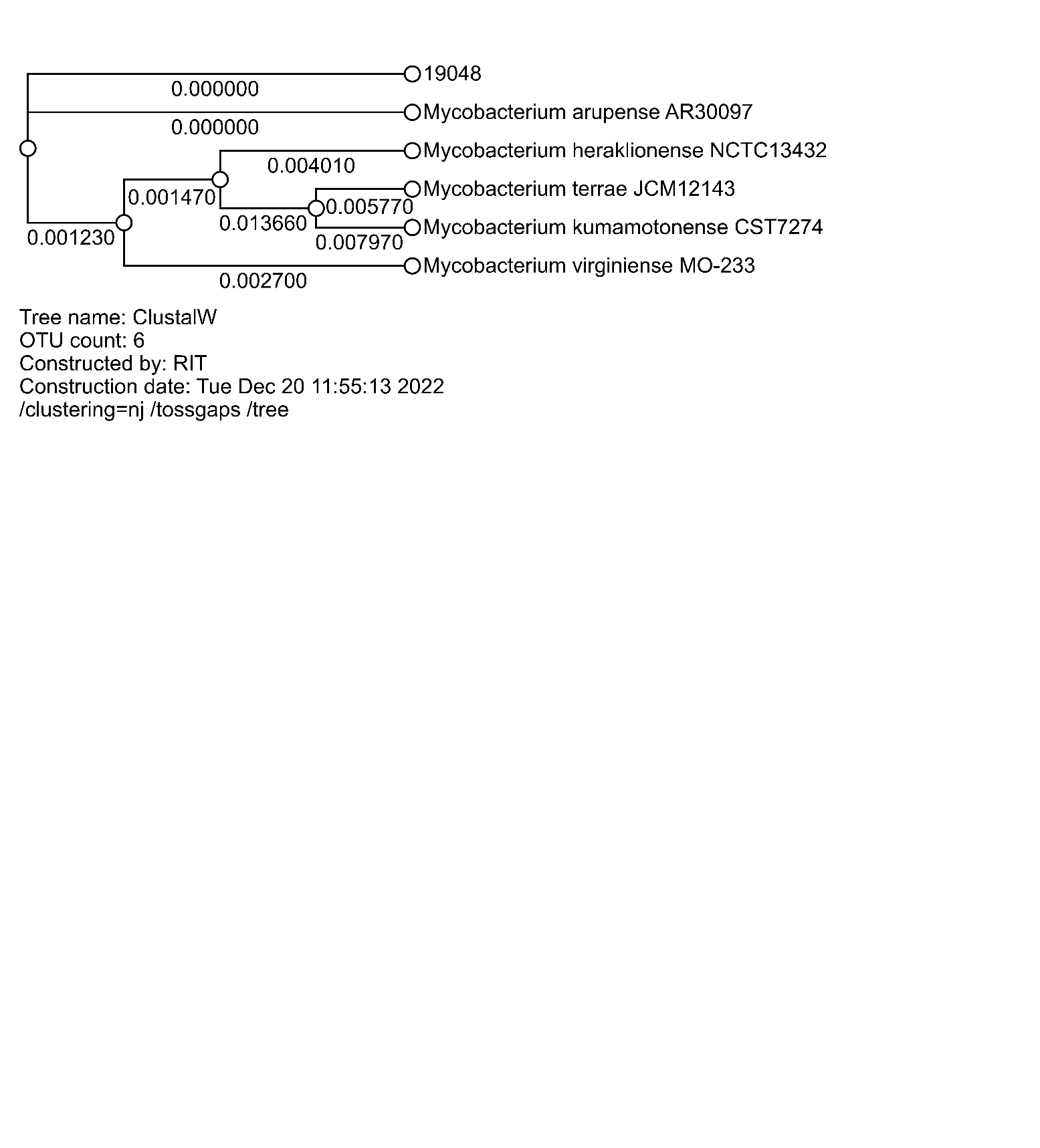
**
